# Thyroid Ultrasound Pitfalls: Esophageal Fibrovascular Polyp Mimicking Thyroid Nodule

**DOI:** 10.1155/2016/3601508

**Published:** 2016-02-28

**Authors:** Anna Ansaloni, G. Brigante, B. Madeo

**Affiliations:** ^1^Unit of Endocrinology, Department of Biomedical, Metabolic and Neural Sciences, University of Modena and Reggio Emilia, Italy; ^2^Azienda USL of Modena, Italy

## Abstract

*Background*. Ultrasound (US) is the most accurate tool in the diagnosis of thyroid nodules if performed by expert physician. Misdiagnosis due to extrathyroidal lesions mimicking thyroid nodules is reported in literature. We describe the first case of an esophageal fibrovascular polyp misdiagnosed as a thyroid nodule on US examination.* Patient Findings*. A 54-year-old woman presented to emergency department for headache and underwent carotid Doppler extended to neck ultrasound with incidental finding of a nodule in the posterior side of the left thyroid lobe. A following thyroid US performed by an endocrinologist allowed the characterization of the lesion as an esophageal pathology, considering the extrathyroidal position, the typical peripheral hyperechoic spots and hypoechoic rim, the connection to the esophagus, and the swallowing connected movement. The patient was addressed to further investigations and finally to anterior pharyngotomy with histological diagnosis of esophageal fibrovascular polyp.* Summary*. Differential diagnosis between thyroid nodules and other neck lesions is important to prevent an unnecessary fine needle aspiration biopsy and to treat the extrathyroidal pathology. In this case, an US performed by an expert endocrinologist allowed detecting an esophageal fibrovascular polyp requiring surgical removal. In conclusion, the possibility of an esophageal pathology, and even fibrovascular polyp, should be considered during US thyroid examination.

## 1. Introduction

Ultrasound (US) scan has a major role in the diagnosis of thyroid nodules. Its accuracy depends mainly on physician experience but some other peculiar conditions may lead to misdiagnosis. Common pitfalls associated with US are related to technical equipment, examination skills, anatomy, interpretation, and extrathyroidal abnormalities [1]. In particular, extrathyroidal lesions misinterpreted as thyroid pathology can be Killian-Jamieson and Zenker diverticula, paratracheal air cysts, parathyroid hyperplasia or adenoma [1], thyroglossal duct cysts, vascular aneurysms, and lymph nodes [2].

Here, we report the first case of an esophageal fibrovascular polyp originally misdiagnosed as a thyroid nodule upon US examination.

## 2. Case Presentation

A 54-year-old Caucasian woman was referred to our unit in March 2014 because of a suspected thyroid nodule. The diagnosis was formulated in the emergency department, where the patient was admitted because of headache, as an incidental finding during US of the carotids and vertebral arteries. The US examination was therefore extended to thyroid; the physician described a unique, solid, hypoechoic nodule, with shell calcification, with maximum diameter of 10 mm, located in the posterior part of the left thyroid lobe.

**Figure 1:**
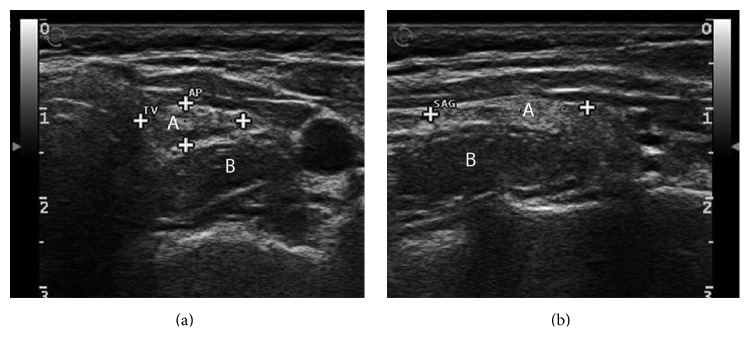
A: transverse (a) and longitudinal (b) ultrasound scans of the left thyroid lobe. B: transverse (a) and longitudinal (b) ultrasound scans of the esophageal lesion outside the posterior pole of the left thyroid lobe.

**Figure 2:**
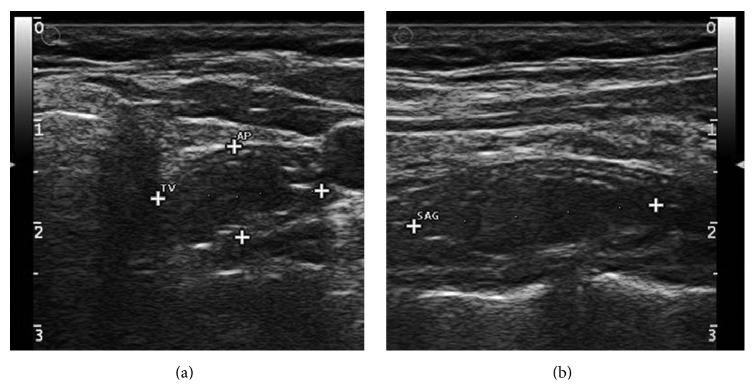
Transverse (a) and longitudinal (b) sonograms show a hypoechoic lesion with internal hyperechoic foci and hypoechoic rim.

**Figure 3:**
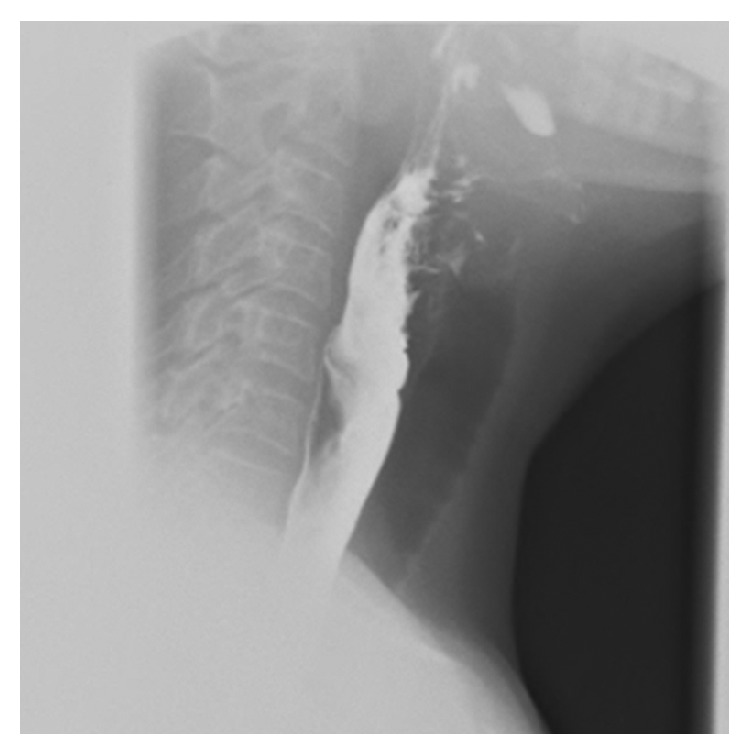
Esophagogram shows a 2 cm oval wall irregularity on the back slope of the distal cervical esophagus.

The patient was on levothyroxine replacement therapy to treat hypothyroidism due to thyroiditis. A second thyroid US was performed by an experienced endocrinologist, using a MyLab25Gold scanner and a linear 5–10-MHz probe (Esaote SpA, Genoa, Italy), showing an atrophic thyroid gland, without nodules (Figure 1). The previously reported thyroid nodule was characterized as an oval, hypoechoic lesion with echogenic foci and peripheral vascularity, measuring 8.9 AP × 15.8 T × 23.4 L mm and located outside of the thyroid capsule, corresponding to an abnormal esophagus (Figure 2). Therefore the patient was referred for esophagography that showed a 2 cm oval wall irregularity on the back slope of the distal cervical esophagus (Figure 3). The patient did not complain about symptoms of dysphagia or pain. The endoscopic exploration confirmed a solid lesion at the level of the upper esophageal orifice. A biopsy examination was performed and a squamous cell papilloma was suspected. Magnetic resonance imaging confirmed the localization and the extension of the mass, while positron emission tomography demonstrated the absence of intralesional metabolic activity. Considering the biopsy, the patient was referred to surgery. The pedunculated lesion was excised through an anterior pharyngotomy. Surprisingly, the final histological diagnosis was fibrovascular polyp of the esophagus. Unluckily, the postoperative course was complicated by the formation of a pharyngoesophageal fistula, treated through a second surgical intervention.

This case report was written according to ethical standards. The patient gave her written consent for publication.

## 3. Discussion

This is the first case report of a misdiagnosis of thyroid nodule due to an esophageal fibrovascular polyp. The differential diagnosis is important to prevent an unnecessary fine needle aspiration biopsy and to refer the patient to a gastroenterologist. Even if US is not the best technique to explore an esophageal lesion, it should be suspected when a mass is seen outside of the thyroid, in most cases posteriorly to the left lobe, and in continuity with the esophagus. Normal esophagus is easily identified thanks to its typical gut sonographic signature, such as hypoechoic mass with internal echogenic dots, peripheral hyperechoic concentric striae, and changing of shape when the patient swallows. However, some abnormalities of the cervical portion may be misdiagnosed as thyroid pathology.

Fibrovascular polyps of esophagus are rare benign tumors of the upper third of the esophagus, burdened by a low detection rate due to the fact that most patients are asymptomatic [3], as in the case presented here. These pedunculated intraluminal masses can demonstrate massive growth, leading to sudden death by block of the larynx and trachea [3]. Due to such potentially disastrous complications, removal of benign esophageal polyps is recommended [4]. Actually, our patient was referred to surgery because of the histological suspicion of a squamous cell papilloma, a lesion with malignant potential [5]. Luckily, the suspicion was not confirmed by the final histological diagnosis. An important aspect of this case is the initial misdiagnosis of thyroid nodule, immediately resolved by performing a targeted thyroid US in experienced hands. Thyroid US should be performed by skilled physicians to better discriminate real thyroid alterations.

In conclusion, the possibility of esophageal pathologies should be considered when a hypoechoic lesion is detected posterior to the left thyroid lobe and in continuity with the esophagus.
